# Characterization of coagulopathy and outcomes in cancer patients with severe COVID ‐19 illness: Longitudinal changes in hospitalized cancer patients

**DOI:** 10.1002/cam4.4753

**Published:** 2022-04-26

**Authors:** Mahsa Madani, Drew Goldstein, Roxana Stefanescu, Scott E. Woodman, Cristhiam M. Rojas‐Hernandez

**Affiliations:** ^1^ The University of Texas McGovern Medical School of Medicine Houston Texas USA; ^2^ Syntropy Technologies LLC Cambridge Massachusetts USA; ^3^ Department of Genomic Medicine, Division of Cancer Medicine The University of Texas MD Anderson Cancer Center Houston Texas USA; ^4^ Section of Benign Hematology The University of Texas MD Anderson Cancer Center Houston Texas USA

**Keywords:** clinical observations, hematological cancer, prognostic factor, risk assessment, survival, viral infection

## Abstract

There is a lack of data focused on the specific coagulopathic derangements in COVID‐19 versus non‐COVID‐19 acutely ill cancer patients. Our objective was to characterize features of coagulopathy in cancer patients with active COVID‐19 illness who required hospitalization at MD Anderson in the Texas Medical Center and to correlate those features with thrombotic complications, critical illness, and mortality within the first 30 days after hospital admission for COVID‐19 illness. COVID‐19 and non‐COVID‐19 hospitalized cancer patients, with at least five consecutive measures of PT, PTT, d‐dimer, and CBC during the same period, were matched 1:1 to perform a retrospective analysis. We reviewed complete blood cell counts with differential, PT, PTT, fibrinogen, D‐Dimer, serum ferritin, IL‐6, CRP, and peripheral blood smears. Clinical outcomes were thrombosis, mechanical ventilation, critical illness, and death. Compared with matched hospitalized cancer patients without COVID‐19, we found elevated neutrophil and lower lymphocyte counts in those with critical illness ( *p =*  0.00) or death ( *p =*  0.00); only neutrophils correlated with thrombosis. COVID‐19 cancer patients with a platelet count decline during the hospital stay had more frequent critical illness ( *p =*  0.00) and fatal outcomes ( *p =*  0.00). Of the inflammatory markers, interleukin‐6 showed consistently higher levels in the COVID‐19 patients with poor outcomes. The findings of unique platelet changes and coagulopathy during severe COVID‐19 illness in the cancer population are of interest to explore disease mechanisms and future risk stratification strategies to help with the management of cancer patients with COVID‐19.

## Graphical abstract

The findings of unique platelet changes and coagulopathic derangements during severe COVID‐19 illness in the cancer population shown in this study would be of interest to explore further disease mechanism and risk stratification strategies to help with management of cancer patients with COVID‐19.

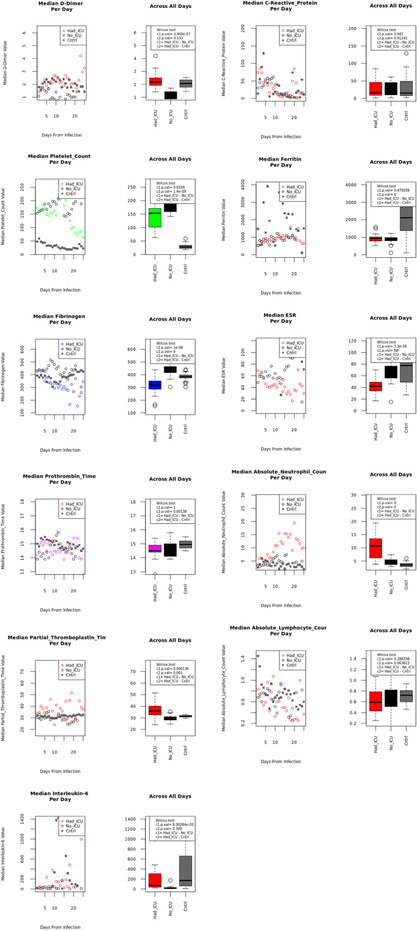

## 1 INTRODUCTION

The COVID‐19 clinical syndrome resulted in remarkable morbidity and deaths worldwide.^1^ Reports show COVID‐19 patients present with several coagulopathic abnormalities: elevated D‐dimer, prothrombin time (PT), and activated thromboplastin time (PTT), with associated intraalveolar fibrin deposition and venous thromboembolism.^2^, ^3^ Multi‐institutional patient data of hospitalized COVID‐19 adults showed that increased D‐dimer, fibrinogen, platelet counts, and inflammatory markers at presentation were predictive of thrombosis and some with critical illness and death.^4^ Tissue and autopsy of COVID‐19 patients showed different mechanisms pertinent to hematologic and vascular systems, that is, endotheliopathy, neutrophil‐TRAPS, and excessive platelet activation.^5^, ^8^

Cancer patients are at high risk for thrombosis and bleeding complications.^9^, ^10^ Cancer patients experience different coagulation parameters and pro‐inflammatory cytokine levels during the course of malignant disease.^11^, ^13^

Our study objectives were to characterize coagulopathic features in cancer patients with active COVID‐19 illness requiring hospital admission at MD Anderson in the Texas Medical Center and to correlate those features with thrombotic complications, critical illness, and mortality within the first 30 days after hospital admission for COVID‐19.

## 2 METHODS

As part of the institutional Data‐Driven Determinants for COVID‐19 Oncology Discovery Effort (D3CODE), IRB‐approved protocol 2020‐0348, COVID‐19 relevant data were analyzed using the Syntropy platform, Palantir Foundry (“Foundry”). All cancer patients consecutively admitted to MD Anderson Hospital in the Texas Medical Center with COVID‐19 between March 19, 2020 and December 22, 2020 were evaluated for study inclusion.

Complete blood cell counts with differential, PT, PTT, fibrinogen, D‐dimer, serum ferritin, interleukin‐6 (IL‐6), and C‐reactive protein (CRP) data were collected and peripheral blood smears (for the presence of microangiopathic anemia) identified for each COVID‐19 patient. We extracted patient demographics and clinical characteristics, in addition to data regarding mechanical ventilation requirements, unit location, and mortality events. Thrombotic outcomes (venous and arterial) were derived from disease ICD‐10 CM codes during 30‐day hospitalization. Initial results looked at whether laboratory parameters were significantly associated with our four clinical outcomes (thrombosis, mechanical ventilation, critical illness, and death) in COVID‐19 cancer patients at initial presentation and its rate of change over a 30‐day hospital course.

Second, we assessed the association between laboratory markers and each clinical outcome in hospitalized cancer patients with COVID‐19 versus without COVID‐19 illness. A 1:1 propensity score matching was performed for age, sex, race, BMI, type of malignancy, and history of diabetes with non‐COVID‐19 hospitalized cancer patients, with a minimum of five consecutive measures of PT, PTT, D‐dimer, and CBC for the same period.

We summarized and compared the distribution of demographic continuous variables using the mean and standard deviation values, laboratory parameters were compared by the median and interquartile range, and the distribution of categorical variables using frequencies of each value. Chi‐squared or Wilcoxon test was used to examine associations between laboratory markers and clinical outcomes of interest. Pearson correlation coefficient was used to assess relationships between individual laboratory markers.

## 3 RESULTS

### 3.1 Patient Characteristics

Our cohort of hospitalized COVID‐19 cancer patients had 300 cases. After 1:1 matching, analysis was restricted to 164 COVID‐19 cancer patients and 164 control cancer cases without COVID‐19.

Baseline characteristics: middle‐aged to elderly, predominantly solid malignancies, and frequent cardiovascular and metabolic comorbidities (Table 1). Thrombotic complications presented in 29 (17.7%) patients, 20 (12.2%) required mechanical ventilation, and 43 (26.2%) died during 30‐day hospital admission.

**TABLE 1:** Baseline characteristics of matched 164 cancer patients with COVID‐19 versus 164 cancer control patients without COVID‐19

Characteristic	Categories/summary statistic	COVID‐19 *N* (%)	Control *N* (%)	*p*
Age (years)	Mean (standard deviation)	59.2 (15.4)	60.4 (16.8)	0.510
Sex	Female	73 (44.5)	73 (44.5)	1.000
Race	White or Caucasian	102 (62.2)	102 (62.2)	1.000
	Black or African American	28 (17.1)	28 (17.1)	
	Asian	7 (4.3)	7 (4.3)	
	Other	26 (15.9)	26 (15.9)	
	Unknown	1 (0.6)	1 (0.6)	
Body mass index	Mean (standard deviation)	30.4 (5.9)	28.2 (5.7)	0.001
Diabetes	Yes	94 (57.3)	94 (57.3)	1.000
Hypertension	Yes	42 (25.6)	42 (25.6)	1.000
Cardiovascular disease	Yes	102 (62.2)	99 (60.4)	0.821
Lung disease	Yes	120 (73.2)	112 (68.3)	0.396
Cancer type	Hematologic	129 (78.7)	129 (78.7)	
	Solid	35	35	
Solid tumor types	Bone	3 (1.8)	8 (4.9)	0.157
	Breast	7 (4.3)	0	
	CNS	3 (1.8)	0	
	CRC	1 (0.6)	5 (3.0)	
	Endocrine	1 (0.6)	3 (1.8)	
	GI	2 (1.2)	2 (1.2)	
	GU	4 (2.4)	3 (1.8)	
	GU, CRC	0	1 (0.6)	
	Gyn	3 (1.8)	2 (1.2)	
	Heme, Breast		1 (0.6)	
	Heme, THN		1 (0.6)	
	HPB	3 (1.8)	3 (1.8)	
	HPB, CRC	1 (0.6)	0	
	SKIN	2 (1.2)	2 (1.2)	
History of bone marrow—HSCT‐transplant	Yes	4 (100.0)	2 (100.0)	1.000
Recent chemotherapy	Yes	108 (65.9)	113 (68.9)	0.638
Recent immunosuppressant	Yes	158 (96.3)	159 (97.0)	1.000
Anticoagulation	Yes	20 (12.2)	112 (68.3)	<0.001
Antiplatelet therapy	Yes	110 (67.1)	143 (87.2)	<0.001

Abbreviations: CRC, colorectal cancer; GI, gastrointestinal; Gyn, gynecological; GU, genitourinary; HPB, hepato‐pancreo‐biliary; THN, thoracic head and neck.

### 3.2 Laboratory parameters and clinical outcomes among COVID ‐19 patients

Among coagulation parameters, we identified higher D‐dimer and lower fibrinogen levels in patients with outcomes of interest (mechanical ventilation and critical illness), while they were not significantly different for thrombosis and death. We observed significantly more prolonged PT in those with poor clinical outcomes (mechanical ventilation and death), but PT prolongation was mild and <3 s in the majority of patients (Table 2).

**TABLE 2:** Comparative description of laboratory parameters in COVID‐19 cancer patients by clinical outcomes: thrombosis, critical illness, and death

Laboratory parametersMedian (IQR)	Normal range	Thrombosis	No thrombosis	*p*	Intensive care	No intensive care	*p*	Mechanical ventilation	No mechanical ventilation	*p*	Death	Survive	*p*
D‐Dimer	0.1–0.5 μg/ml	2.2 [1.6–2.4]	1.7 [1.6–2.2]	0.24	2.2 [1.9–2.5]	1.0 [0.9–1.4]	0.00	2.9 [2.7–4.4]	1.4 [1.3–1.6]	0.00	1.9 [1.7–2.1]	1.7 [1.6–2.3]	0.44
Fibrinogen	214–503 mg/dl	368 [323–422]	402 [288–453]	0.71	320 [287–351]	438 [417–465]	0.00	284 [238–345]	428 [393–449]	0.00	351 [166–400]	391 [338–427]	0.08
PT	12–14.3 s	14.6 [14.3–14.8]	14.5 [14.2–15.0]	0.71	14.5 [14.4–14.9]	14.5 [14.1–15.0]	1.00	14.1 [13.9–14.5]	14.7 [14.4–14.9]	0.00	16.1 [15.2–16.7]	14.4 [14.1–14.5]	0.00
PTT	24.2–36 s	32.7 [29.5–39.0]	34.2 [27.2–35.6]	0.69	35.8 [32.5–40.1]	29.7 [28.1–30.9]	0.00	35.3 [28.5–44.4]	33.7 [29.7–35.0]	0.71	35.7 [32.8–43.3]	33.1 [28.6–35.6]	0.03
ANC	1.7–7.3 ×10^3^/μl	7.3 [4.7–10.1]	8.6 [4.8–14.5]	0.21	10.7 [6.2–13.5]	4.6 [3.7–5.5]	0.00	11.4 [9.7–15.4]	5.7 [4.3–7.2]	0.00	9.9 [7.0–13.9]	6.4 [4.4–8.9]	0.00
ALC	1.0–4.8 ×10^3^/μl	0.8 [0.7–0.9]	0.5 [0.3–0.7]	0.00	0.6 [0.4–0.8]	0.7 [0.5–0.8]	0.28	1.0 [0.5–1.3]	0.6 [0.3–0.8]	0.00	0.4 [0.3–0.6]	0.7 [0.5–0.8]	0.01
Platelet count	140–440 ×10^3^/μl	163 [142–174]	164 [102–180]	0.86	153 [102–171]	183 [159–196]	0.01	145 [99–182]	176 [161–191]	0.08	143 [88–170]	178 [147–196]	0.03
IL‐6	0–5 pg/ml	162.0 [27.5–896.0]	48.0 [24.8–70.5]	0.10	70.0 [37.0–307.0]	14.5 [10.0–29.0]	0.00	67.5 [44.0–391.0]	20.5 [12.0–132.5]	0.01	47.0 [36.0–84.5]	141.5 [20.0–482.0]	0.34
Ferritin	30–400 ng/ml	878.2 [746.0–1094.0]	849.0 [762.0–1383.0]	0.58	933.0 [785.5–1015.0]	866.5 [799.0–980]	0.47	1071.0 [933.0–1186.0]	823.0 [743.0–950.0]	0.00	934.0 [773.0–1494.0]	967.0 [738.0–1089.0]	0.38
CRP	<10.0 mg/L	30.3 [10.1–42.9]	5.2 [2.2–38.2]	0.05	16.0 [9.0–46.0]	16.8 [10.1–45.5]	0.94	11.2 [3.0–46.6]	15.8 [7.5–52.8]	0.51	16.2 [2.2–47.8]	28.0 [11.9–41.7]	0.25
ESR	0–9 mm/h	44.5 [37.5–51.0]	55.0 [50.0–66.0]	0.02	42.0 [34.0–49.0]	62.5 [56.0–76.5]	0.00	39.5 [30.0–48.0]	57.5 [52.5–60.7]	0.00	49.0 [37.0–59.0]	50.0 [41.0–55.0]	0.98

Abbreviation: IQR, interquartile range.

Blood counts showed consistently an elevated neutrophil among patients with critical illness or death, while the lymphocyte count was significantly lower in those who died. Across the subgroups of clinical outcomes, lymphopenia was common in cancer patients with COVID‐19. However, there was no correlation between those same leukocyte count trends with thrombosis (Table 2). Platelet count was normal in most patients and lower platelet counts were seen with critical illness and fatal outcomes (Appendix A).

Among the serum inflammatory markers, elevated IL‐6 were consistently elevated in patients with mechanical ventilation and critical illness outcomes (Table 2). The sedimentation rate was consistently elevated among patients with cancer and COVID‐19 while less pronounced in those with thrombosis and critical illness outcomes.

### 3.3 Peripheral blood smear findings

We evaluated the first 175 admitted COVID‐19 cancer patients' peripheral blood smears for microangiopathy and found 24/175 with schistocytes. All were mild (<5 per hpf).

### 3.4 Comparison with non‐COVID hospitalized cancer patients

Coagulopathic abnormalities were observed in both COVID‐19 and other ill cancer patients. Both populations had elevated D‐dimer; however, levels were higher in other cancer‐related complications. Notably, the PT was mildly prolonged in both groups (<3 s) and at a lower degree in COVID‐19 patients, except for those who experienced death during the hospitalization (Table 3).

**TABLE 3:** Comparative description of laboratory parameters in COVID‐19 cancer patients versus control hospitalized cancer population without COVID‐19

Laboratory parametersMedian (IQR)	Normal range	Thrombosis	Control	*p*	Intensive care	Control	*p*	Mechanical ventilation	Control	*p*	Death	Control	*p*
D‐Dimer	0.1–0.5 μg/ml	2.2 [1.6–2.4]	2.0 [1.8–2.3]	0.96	2.2 [1.9–2.5]	2.0 [1.8–2.3]	0.13	2.9 [2.7–4.4]	2.0 [1.8–2.3]	0.00	1.9 [1.7–2.1]	2.0 [1.7–2.2]	0.41
Fibrinogen	214–503 mg/dl	368 [323–422]	387 [362–398]	0.75	320 [287–351]	386 [364–406]	0.05	284 [238–345]	387 [362–398]	0.00	351 [166–400]	375 [343–393]	0.29
PT	12–14.3 s	14.6 [14.3–14.8]	15.1 [15.0–15.2]	0.00	14.5 [14.4–14.9]	14.9 [14.7–15.2]	0.00	14.1 [13.9–14.5]	15.1 [15.0–15.2]	0.00	16.1 [15.2–16.7]	14.8 [14.7–15.1]	0.00
PTT	24.2–36 s	32.7 [29.5–39.0]	31.7 [31.0–32.3]	0.25	35.8 [32.5–40.1]	31.3 [30.7–32.0]	0.00	35.3 [28.5–44.4]	31.7 [31.0–32.3]	0.12	35.7 [32.8–43.3]	30.9 [30.1–31.6]	0.00
ANC	1.7–7.3 ×10^3^/μl	7.3 [4.7–10.1]	3.4 [3.1–3.9]	0.00	10.7 [6.2–13.5]	3.5 [2.8–4.0]	0.00	11.4 [9.7–15.4]	3.4 [3.0–3.9]	0.00	9.9 [7.0–13.9]	3.3 [2.5–3.7]	0.00
ALC	1.0–4.8 ×10^3^/μl	0.8 [0.7–0.9]	0.7 [0.6–0.8]	0.21	0.6 [0.4–0.8]	0.7 [0.6–0.8]	0.06	1.0 [0.5–1.3]	0.7 [0.6–0.8]	0.34	0.4 [0.3–0.6]	0.7 [0.5–0.8]	0.00
Platelet count	140–440 ×10^3^/μl	163 [142–174]	26 [23–31]	0.00	153 [102–171]	28 [23–34]	0.00	145 [99–182]	26 [23–31]	0.00	143 [88–170]	27 [24–32]	0.00
IL‐6	0–5 pg/ml	162.0 [27.5–896.0]	134.5 [57.0–660.0]	0.91	70.0 [37.0–307.0]	171.0 [61.0–660.0]	0.30	67.5 [44.0–391.0]	134.0 [57.0–660.0]	0.43	47.0 [36.0–84.5]	171.0 [61.0–660.0]	0.05
Ferritin	30–400 ng/ml	878.2 [746.0–1094.0]	2512.0 [1518.0–3434.0]	0.00	933.0 [785.5–1015.0]	2117.0 [1376.0–3374.0]	0.00	1071.0 [933.0–1186.0]	2512.0 [1518.0–3434.0]	0.00	934.0 [773.0–1494.0]	2512.0 [1469.5–3434.0]	0.00
CRP	<10.0 mg/L	30.3 [10.1–42.9]	29.7 [12.1–64.3]	0.51	16.0 [9.0–46.0]	15.5 [8.0–49.2]	0.91	11.2 [3.0–46.6]	29.7 [12.1–64.3]	0.05	16.2 [2.2–47.8]	13.9 [6.8–51.2]	0.35
ESR	0–9 mm/h	44.5 [37.5–51.0]	41.0 [27.0–67.0]	0.46	42.0 [34.0–49.0]	41.0 [27.0–67.0]	0.79	39.5 [30.0–48.0]	41.0 [27.0–67.0]	0.79	49.0 [37.0–59.0]	34.0 [25.0–71.0]	0.42

Abbreviation: IQR, interquartile range.

Blood counts showed the most remarkable differences. Platelet and neutrophil counts were consistently higher in COVID‐19 cancer patients, which contrasted with lower lymphocyte counts across groups, predominantly in patients with COVID‐19 who died (Table 3) (Appendix A).

Similar to the PT and D‐dimer levels, inflammatory markers were nonspecifically elevated in both groups; although serum ferritin levels in COVID‐19 cases were high, the increment was of lower magnitude than in other ill cancer patients.

## 4 DISCUSSION

Common coagulopathic findings in patients with COVID‐19 may be altered in the cancer population who have elevated inflammatory markers and derangements in coagulopathic parameters at baseline.

One study by Patell et al. looked at the incidence of thrombotic and hemorrhagic events in those with cancer and COVID‐19 versus those without cancer. They found a similarly high incidence of thrombosis and bleeding events among cancer and noncancer patients with COVID‐19.^14^ Platelet counts in COVID‐19 cancer patients were found to have similar median parameters to COVID‐19 cancer patients in our study. Additionally, unlike Patell et al., our study examined cancer patients without COVID‐19, and we observed that at baseline, these patients presented with more severe thrombocytopenia compared to cancer patients with acute COVID‐19 illness.

Al‐Samkari et al. study looked at bleeding and thrombotic manifestations of COVID‐19 in patients without cancer. They found thrombocytopenia predictive of bleeding and thrombotic complications.^4^ Our cohort had a unique coagulopathic characterization. Although platelet levels were commonly within the normal range (compared with non‐COVID hospitalized cancer controls), those COVID‐19 patients who experienced profound platelet count decline during hospital stay (Table 1) had more thrombotic events, critical illness, and fatal outcomes.

Several hypotheses may explain our observations. One study reported that during acute COVID‐19 illness, there are platelet changes related to P‐selectin, an adhesion receptor on activated platelets for leukocytes, that has been shown as a player in tumor metastasis,^8^ but also hyperactivated in noncancer COVID patients. This has been studied as a possible factor in the hypercoagulability seen in COVID‐19 infection.^8^ Thrombocytopenia secondary to platelet consumption during disseminated intravascular coagulation (DIC) is observed in critically ill cancer patients, however, the classic changes of DIC—fibrinogen consumption and several fold‐prolongation of prothrombin time—were not seen in our cohort. Moreover, we did not observe microangiopathic changes in most peripheral blood smears obtained from the majority of our patients.

As described in noncancer patients, elevated neutrophil counts, depleted lymphocyte counts, elevated D‐dimer levels, and serum inflammatory markers (e.g., IL‐6) were associated with poor clinical outcomes in our cancer population. The platelet count changes seen in our hospitalized cases of COVID‐19 with a cancer diagnosis and their correlation with clinical outcomes are unique compared with other COVID‐19 hospitalized populations.

Interactions between endothelium and platelets during severe COVID‐19 illness may explain some changes seen in the platelet and coagulation parameters.^15^ Additional studies may elucidate the unique pathophysiology effects of COVID‐19 on the vascular and hematologic systems.

The strengths of our study include the longitudinal and rich sample of coagulation and inflammatory biomarkers in a cohort of consecutively admitted cancer patients with COVID‐19. We evaluated for associations between thrombotic complications, hard clinical outcomes (mortality and mechanical ventilation) and the laboratory parameters; then compared those with a control cohort of hospital‐admitted cancer patients without COVID‐19. Although we incorporated the type of malignancy and administration of recent chemotherapy, our analyses did not explore associations between specific types of chemotherapy, particularly lymphodepleting therapies (i.e., cyclophosphamide, fludarabine, bendamustine, and others) which may have had a significant impact in the outcomes described in our manuscript. This represents an important limitation to our conclusions. We attempted to mitigate the heterogeneity between the COVID‐19 and the non‐COVID‐19 cohorts during the analyses by performing a 1:1 propensity score matching for age, sex, race, BMI, type of malignancy, and history of diabetes. Additionally, we could not perform subgroup analyses by different tumor primary sites, which could have also impacted the outcomes and limited the extrapolation of our observations.

In conclusion, our observations on the coagulopathy and unique platelet count changes during severe COVID‐19 illness in the cancer population are of interest to explore mechanisms of disease and may help develop future disease severity risk stratification strategies.

## CONFLICT OF INTEREST

None.

## AUTHOR CONTRIBUTION

Dr. Cristhiam M. Rojas‐Hernandez devised the presented idea. Dr. Mahsa Madani took lead in writing the manuscript with the support of Dr. Cristhiam M. Rojas‐Hernandez, Mr. Drew Goldstein, and Dr. Scott E. Woodman. Mr. Drew Goldstein helped with the computational framework and analysis of the data. All authors provided critical feedback and helped shape the research, analysis, and manuscript.

## ETHICS APPROVAL STATEMENT

IRB‐approved and D3CODE protocol ID 2020‐0348.

## Data Availability

The data that support the findings of this study are available from the corresponding author upon reasonable request.

## APPENDIX A

### A.1

Longitudinal trends of coagulation, platelet counts, serum inflammatory biomarkers, and leukocyte counts during 30 days following hospitalization for COVID‐19 in 164 cancer patients and comparison with 164 hospitalized cancer patients without COVID‐19

1A. Comparison by thrombosis outcome

1B. Comparison by intensive care outcome

1C. Comparison by mechanical ventilation outcome

1D. Comparison by the fatal outcome
